# Abdominal Imaging Findings in Laboratory-Confirmed COVID-19 Patients: A Retrospective Observational Study

**DOI:** 10.7759/cureus.42677

**Published:** 2023-07-30

**Authors:** Tareq Al Taei, Zainab Yusuf, Sarah Al Mail, Fatema Bunajem, Bedor Abdulrahim, Marwa Meshkhas, Khaled Sulail

**Affiliations:** 1 Radiology, Salmaniya Medical Complex, Manama, BHR

**Keywords:** ultra-sonography, gastro-intestinal, gall blader disease, abdominal pain, covid-19

## Abstract

Background and aim: Coronavirus disease 2019 (CO­VID-19) is known to predominantly present with respiratory symptoms; however, a significant proportion of patients present with digestive symptoms. These symptoms are often non-specific and as such prompt the treating physician to request further imaging evaluation. Understanding the abdominal imaging findings in COVID-19 and their possible associations is thus crucial to direct patient care and prevent misdiagnosis. The aim of this study was to describe abdominal imaging findings on both computed tomography (CT) and ultrasound scans in cases with positive COVID-19 polymerase chain reaction (PCR) tests performed at our institution, and also, to evaluate the reason for requesting these imaging studies, and to correlate these findings with patients’ demographics.

Methods: A retrospective observational study was conducted at Salmaniya Medical Complex, Bahrain, between February 2021 and March 2022. We examined the abdominal CT and ultrasound data for PCR-confirmed COVID-19 patients. The demographic data, reason for requesting imaging and imaging findings were gathered by reviewing the hospital’s electronic health records and picture archiving and communicating system (PACS).

Results: The study included 97 patients, with the majority being male (57.7%). The most common reason for imaging was abdominal pain, as seen in over half of the patients (60.8%), followed by deranged liver enzymes (18.6%). More than 75% of imaging studies showed positive abdominal findings with the majority (19.6%) showing non-specific inflammatory findings, followed by gallbladder disease (13.4%). The CT studies were more likely to yield positive findings as compared to ultrasound, with only 7 (11.3%) CT scans yielding normal findings (χ^2^ = 14.65; P < 0.01).

Conclusion: To our knowledge, the research conducted on the abdominal manifestations of COVID-19 is still limited, especially in our region. Our study showed that there are variable presentations of abdominal organ involvement in COVID-19 cases, and as such more data is required to direct choice of imaging study, protocol, and interpretation of findings to better guide patient management.

## Introduction

Novel coronavirus disease 2019 (COVID-19), caused by severe acute respiratory syndrome coronavirus 2 (SARS-CoV-2), was officially declared a global pandemic by the World Health Organization on March 11, 2020 [1]. Since then, the COVID-19 pandemic has rapidly spread across the globe with over 767 million confirmed cases as of June 2023 and a reported death toll of almost seven million [2]. As the world continues to grapple with the effects of COVID-19, there has been a growing interest in further understanding the various clinical manifestations associated with it. While COVID-19 is primarily known for being a respiratory disease, studies have shown that a significant number of patients present with gastrointestinal symptoms [3,4].

It has been established that angiotensin-converting enzyme 2 (ACE2) receptors, the functional receptor for SARS-CoV-2, play a critical role in the pathogenesis of COVID-19 by facilitating the virus's entry into human cells [5]. ACE2 exhibits its highest levels of surface expression on lung alveolar epithelial cells and in the enterocytes of the small intestine [5]. This suggests one of the possible mechanisms in which the abdominal viscera may be prone to injury.

Previous radiological studies on COVID-19 patients have primarily focused on pulmonary imaging modalities, such as chest CT, and common findings such as ground glass opacities, consolidations, and air bronchograms have become well-established among radiologists [6,7]. However, despite the evidence that COVID-19 can also affect the gastrointestinal system, there has not been any corresponding abdominal imaging findings reported yet. The purpose of this study is to better understand abdominal imaging findings in patients with COVID-19 and to explore possible associations to achieve a better understanding of the abdominal phenomena of COVID-19.

## Materials and methods

The abdominal CT and ultrasound data were retrospectively reviewed over a one-year period between February 2021 and March 2022 at Salmaniya Medical Complex, Kingdom of Bahrain. The demographic data, reason for requesting imaging and imaging findings were collected by reviewing the hospital’s electronic health records and picture archiving and communicating system (PACS). Patients with a diagnosis of laboratory-confirmed SARS-CoV-2 infection who were admitted to our hospital at the time of the scan were included in the study. Patients who tested negative for COVID-19 and were not hospitalized at the time of the scan were excluded. Ethical approval from the Institutional Review Board (IRB) at Salmaniya Medical Complex was obtained before commencing the study (registration no. 2022-511).

The data were collected and compiled in Microsoft Excel, version 2019 (Microsoft Corporation, Redmond, WA). After checking for completeness and consistency, data were analyzed using IBM SPSS for Windows, version 26 (IBM Corp., Armonk, NY). Categorical variables, presented as percentages and frequency distribution, were compared using the chi-square or Fisher’s exact tests. Continuous variables, presented as means and standard deviations, were compared using the Student's independent t-test and analysis of variance (ANOVA). Figures were used to provide an illustrative summary of data. The significance level was defined as α = 0.05. All the ultrasound examinations were scanned by a certified sonographer using GE Lunar (GE Healthcare, Chicago, IL). The CT scans were performed on multidetector CT (MDCT) scanners (GE Healthcare, and Siemens, Erlangen, Germany). Abdominal CT scans were acquired at a 2 mm slice thickness and reconstructed as 5 mm axial slices. The scans were reviewed by two body imaging consultant radiologists with >10 years of experience.

## Results

Participant characteristics

**Figure 1:**
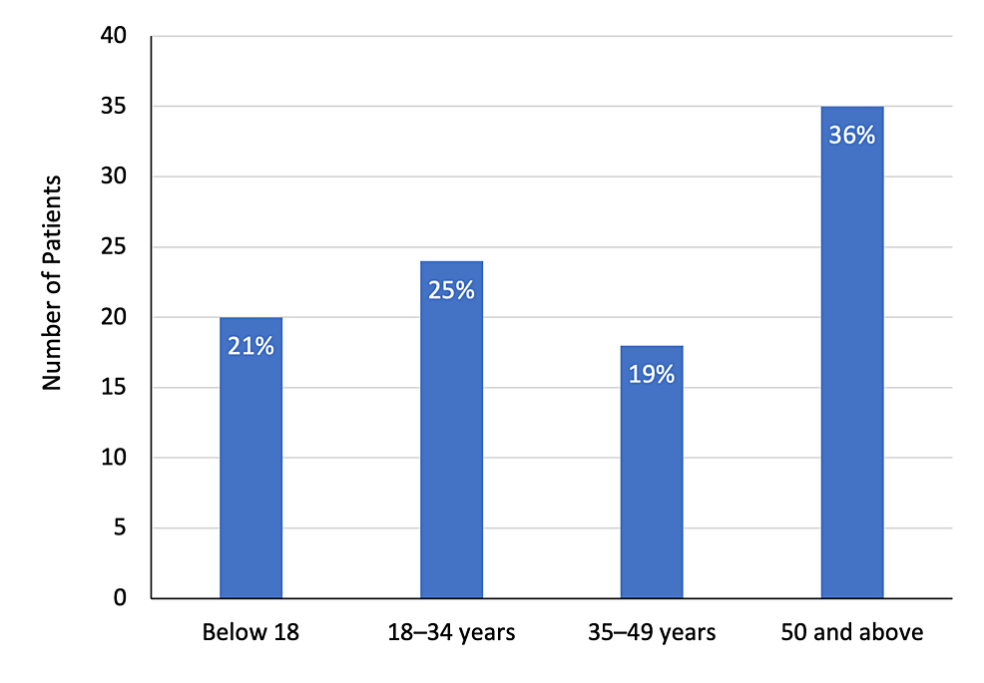
Age distribution of patients

**Figure 2:**
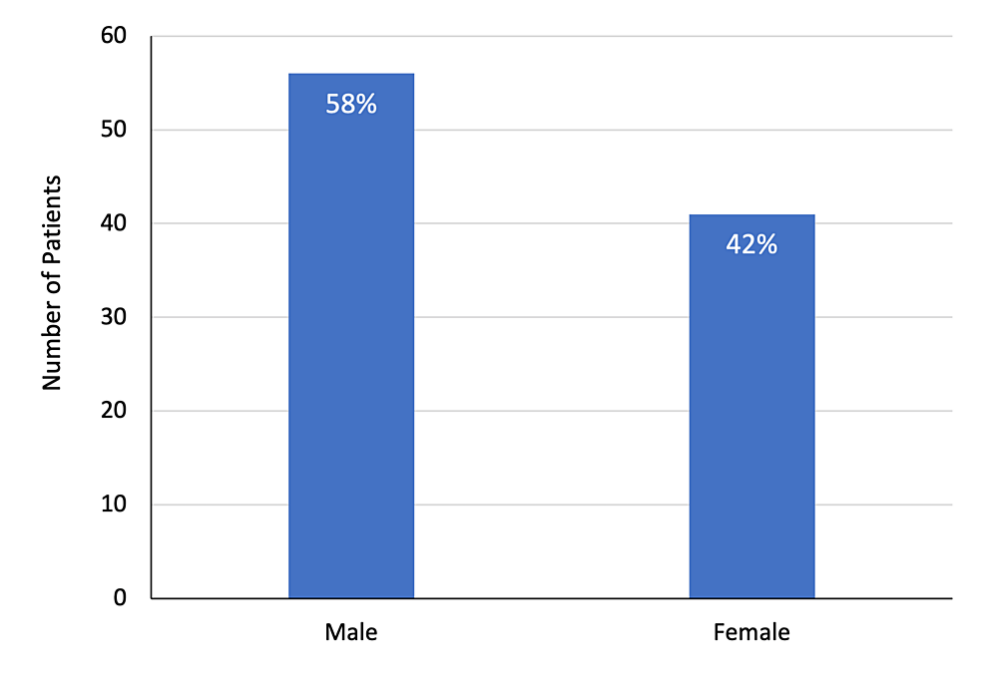
Gender distribution of patients

**Figure 3:**
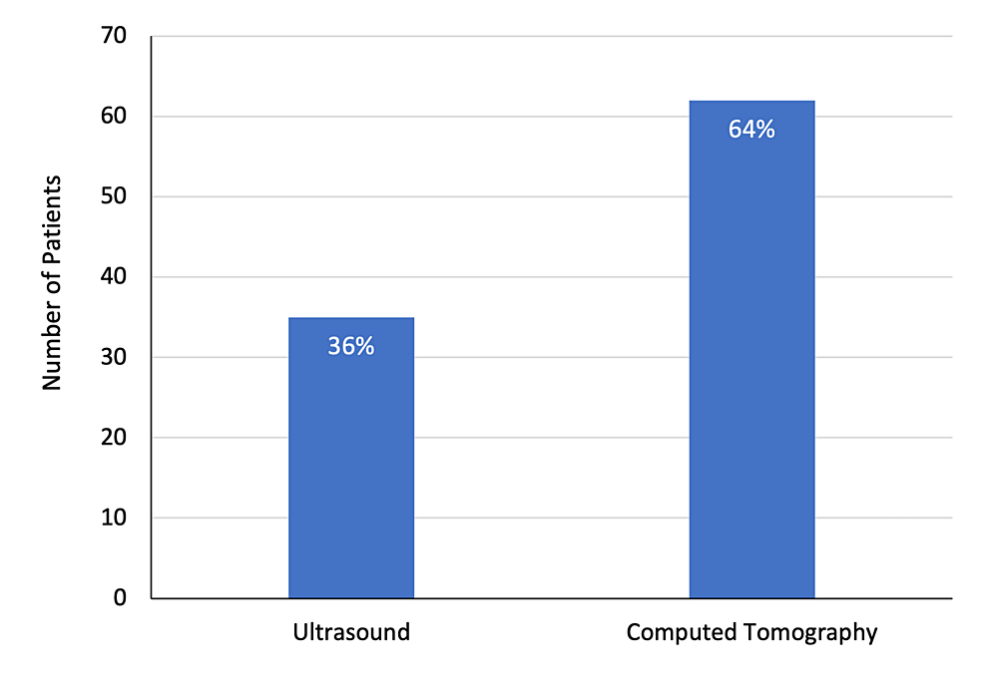
Imaging modalities performed for patients

The study included 97 patients, including 56 (57.7%) men and 41 (42.2%) women. The mean age of patients was 40.9 ± 22.7 years. In total, 22 (35.5%) patients aged 50 years and above, comprising the largest proportion of the study group. Furthermore, 20 (20.6%) patients were below the age of 18 years. Overall, 35 (36.1%) patients underwent abdominal ultrasound examination and the remaining 62 (63.9%) had a CT scan of the abdomen (Figures 1-3).

Indications for imaging studies

The patients underwent abdominal imaging studies for different indications (Table 1). Overall, the most common reason for imaging was abdominal pain, as seen in over half of the patients (60.8%). The right iliac fossa was the most common site of abdominal pain, reported by 23 (39.7%) patients. Other patients reported the abdominal pain in the flank region (19.0%), suprapubic (10.3%), and epigastric area (6.9%). Furthermore, 12 (12.4%) patients had generalized abdominal pain.

**Table 1:** Indications for abdominal imaging studies

Indication	N	%
Abdominal pain	59	60.8
Right iliac fossa	23	23.7
Left iliac fossa	2	2.1
Suprapubic	6	6.2
Flank	11	11.3
Epigastric	4	4.1
Generalized/unspecified	12	12.4
Deranged liver enzymes	18	18.6
Abdominal distension	4	4.1
Miscellaneous	16	16.5

In total, 18 (18.6%) patients had elevated liver enzymes, being the second most common indication of abdominal imaging in the study group. Only 4 (4.1%) patients had abdominal distension. There were 16 patients who underwent imaging for other miscellaneous indications, including recurrent vomiting, unexplained anemia, suspected intussusception, and possible intra-abdominal collection.

Findings of imaging studies

Out of the 97 abdominal imaging studies, 23 (23.7%) studies were considered normal with no identified findings to explain the abdominal symptoms (Table 2). In total, 19 (19.6%) imaging studies had non-specific inflammatory findings, including lymphadenopathy, free abdominal or pelvic fluid, and mesenteric edema. Furthermore, 8 (8.2%) patients were found to have acute appendicitis and 13 (13.4%) patients had gallbladder disease. Ureteric stones were identified in 7 (7.2%) patients. Other findings included acute diverticulitis, acute pyelonephritis, and ileus.

**Table 2:** Findings of abdominal imaging studies

Indication	N	%
Normal findings	23	23.7
Acute appendicitis	8	8.2
Ureteric stones	7	7.2
Non-specific inflammatory changes	19	19.6
Gallbladder disease	13	13.4
Miscellaneous	27	27.8

Factors associated with imaging findings

**Figure 4:**
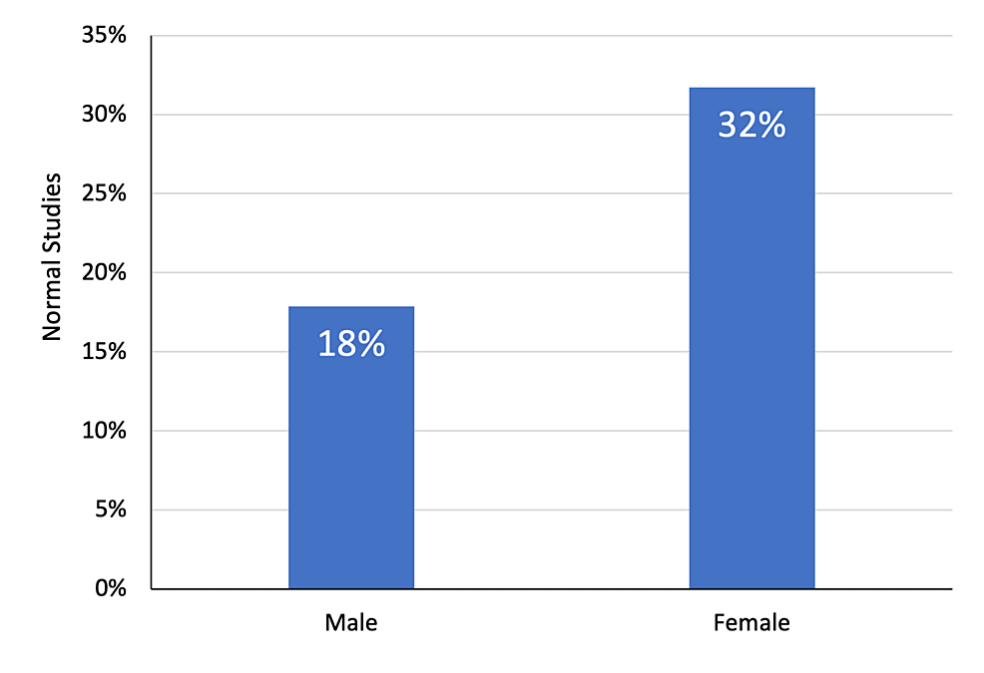
Imaging findings in relation to gender of patients

**Figure 5:**
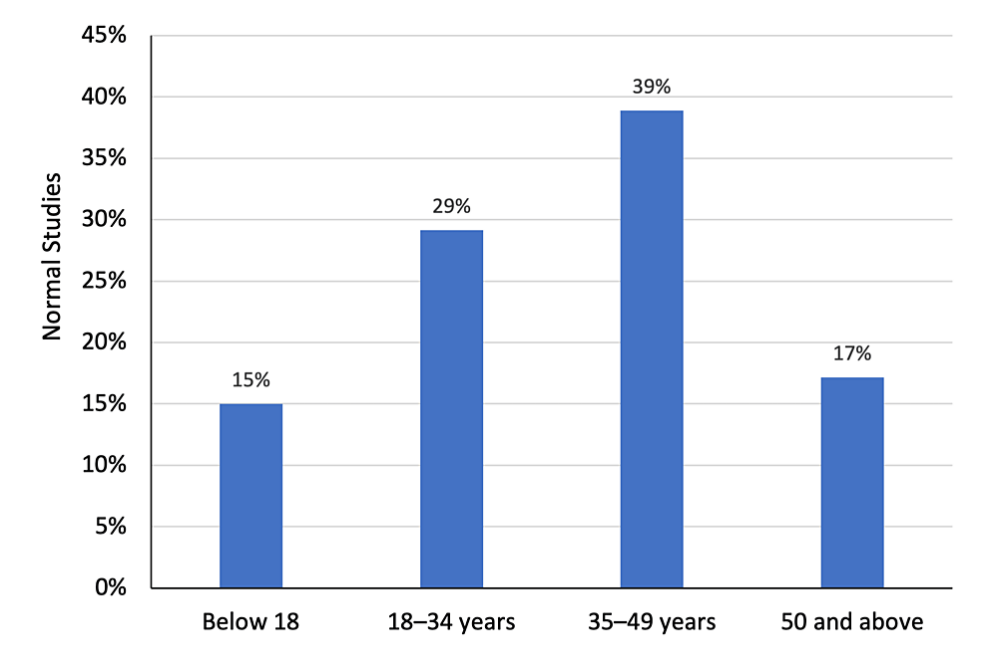
Imaging findings in relation to age of patients

**Figure 6:**
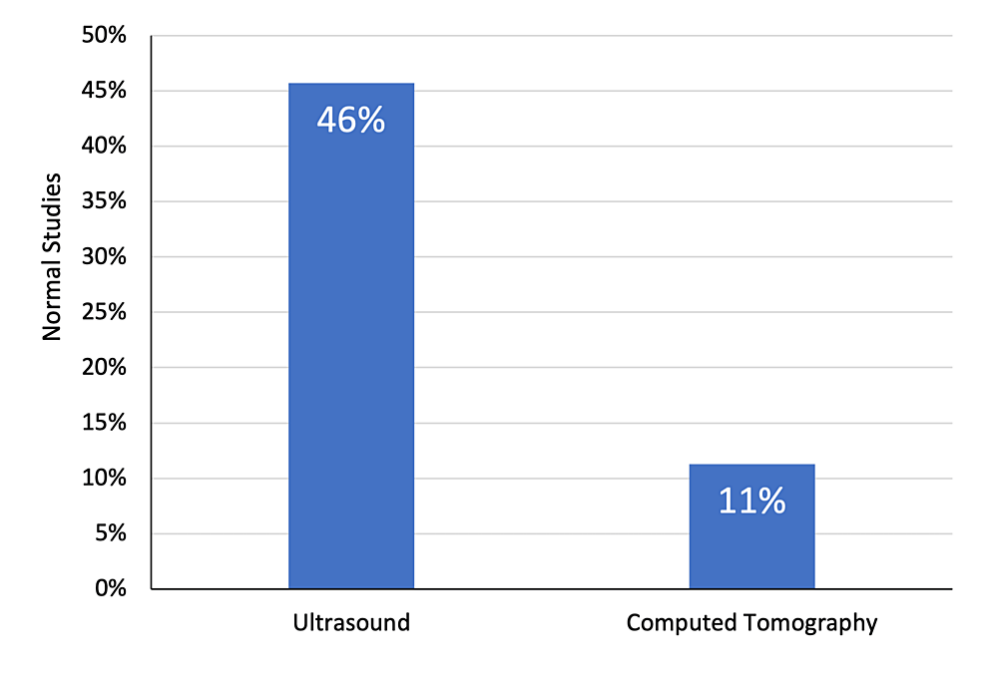
Imaging findings in relation to imaging studies

Overall, 13 (31.7%) female patients had normal abdominal imaging findings compared with 10 (17.9%) males. However, the difference was not statistically significant (χ^2^ = 2.51; P = 0.11). Furthermore, the mean age of patients with normal study findings (37.9 ± 18.2 years) was comparable to those having abnormal imaging findings (41.8 ± 24.0 years) (P = 0.41). The ultrasound studies were more likely to yield no abnormalities. In particular, 16 (45.7%) ultrasound examinations were considered normal while only 7 (11.3%) CT scans yielded normal findings (χ^2^ = 14.65; P < 0.01) (Figures 4-6).

## Discussion

Abdominal signs and symptoms in patients with COVID-19 have been well-documented in multiple studies [3,4]. One study conducted showed that 50.5% of COVID-19 patients presented with gastrointestinal complaints such as abdominal pain, vomiting, and diarrhea [8]. A potential mechanism that could explain this phenomenon is the pattern of ACE2 receptor expression. ACE2 receptors act as the functional host for SARS-CoV2, granting the virus entry into human cells [5]. In the gastrointestinal tract, ACE2 is highly expressed in both the small intestine and the colon. In particular, the epithelial absorptive enterocytes of the ileum have shown high levels of expression [9]. This suggests that the gastrointestinal tract may be susceptible to direct infection by the virus.

In our study, abdominal pain was the most common reason for requesting imaging, accounting for 60.2% of cases; this was followed by deranged liver enzymes in 18.6% of cases. The exact nature of the relationship between COVID-19 infection and hepato-biliary dysfunction remains unclear; however, the presence of ACE2 receptor expression in the biliary system provides a potential association [10]. A retrospective study conducted in Shanghai revealed that 37.2% of COVID-19-positive patients were found to have abnormal liver function tests upon hospital admission [11]. Imaging findings suggestive of gallbladder disease were present in 13.6% patients of our study population. Findings such as gallbladder distension, gallbladder wall thickening, and mural edema have previously been reported in other studies [12-14].

This study has some limitations that should be considered. First, the retrospective design may have limited the completeness of data collection, as we were only able to review the imaging studies and not follow up with the patients to assess for any changes or progression in the findings. In addition, our study did not have a comparison group with non-COVID patients with similar complaints, which limited our ability to draw conclusions about the specificity of findings in COVID patients.

## Conclusions

In conclusion, our study provides valuable insights into the abdominal imaging findings in COVID-19 patients and highlights the need for further research in this area. Our study suggests that there are variable presentations of abdominal involvement in COVID-19 and additional studies are required to further evaluate the topic.
